# Polydopamine/SWCNT Ink Functionalization of Silk Fabric to Obtain Electroconductivity at a Low Percolation Threshold

**DOI:** 10.3390/ijms25095024

**Published:** 2024-05-04

**Authors:** Anna Baranowska-Korczyc, Dorota Kowalczyk, Małgorzata Cieślak

**Affiliations:** Łukasiewicz Research Network–Lodz Institute of Technology, Department of Chemical Textiles Technologies, 9/27 M. Skłodowskiej-Curie Street, 90-570 Lodz, Poland

**Keywords:** silk, silk fabric, SWCNT, conductive ink, conductivity, PDA, polydopamine

## Abstract

This study presents the functionalization of silk fabric with SWCNT ink. The first step was the formation of a polydopamine (PDA) thin coating on the silk fabric to allow for effective bonding of SWCNTs. PDA formation was carried out directly on the fabric by means of polymerization of dopamine in alkali conditions. The Silk/PDA fabric was functionalized with SWCNT ink of different SWCNT concentrations by using the dip-coating method. IR and Raman analyses show that the dominant β-sheet structure of silk fibroin after the functionalization process remains unchanged. The heat resistance is even slightly improved. The hydrophobic silk fabric becomes hydrophilic after functionalization due to the influence of PDA and the surfactant in SWCNT ink. The ink significantly changes the electrical properties of the silk fabric, from insulating to conductive. The volume resistance changes by nine orders of magnitude, from 2.4 × 10^12^ Ω to 2.3 × 10^3^ Ω for 0.12 wt.% of SWCNTs. The surface resistance changes by seven orders of magnitude, from 2.1 × 10^12^ Ω to 2.4 × 10^5^ Ω for 0.17 wt.% of SWCNTs. The volume and surface resistance thresholds are determined to be about 0.05 wt.% and 0.06 wt.%, respectively. The low value of the percolation threshold indicates efficient functionalization, with high-quality ink facilitating the formation of percolation paths through SWCNTs and the influence of the PDA linker.

## 1. Introduction

In recent years, the significant progress in studies on conductive inks has been noticed. Conductive inks based on carbon nanotubes (CNT)s, graphene, silver nanowires (AgNWs) or silver nanoparticles (AgNPs) have been introduced, with some of them becoming commercial products, which has additionally increased the interest in their application and in the design of smart e-textiles [1]. Smart textiles obtained using conductive inks can react, feel and communicate because they are electroconductive.

Owing to their unique material properties related to the one-dimensional nature of electron transport, SWCNTs have been proposed for the fabrication of various conductive inks designed for further electronic applications, with great potential especially for flexible electronics [2,3].

SWCNT ink makes the polyester fabric highly conductive, demonstrating electromagnetic interference shielding [4]. Semiconducting SWCNT inks have been applied to design highly reproducible Field Effect Transistors (FETs) [5]. Functionalized carbon nanotube/water ink characterized by semiconducting behavior and tunable electrical properties was printed on paper to form simple high-frequency electronic devices such as resistances, capacitances or inductances, with values that can be changed in a controllable manner by an applied dc voltage [6]. The first report on the fabrication of flexible thermoelectric generators (TEG) by printing on a curved surface (a bracelet-type TEG structure) also applied CNT ink. [7]. There are many reports on the usefulness of CNT ink in sensor and biosensor design. SWCNT ink has been applied for writing on cellulose paper and designing an ammonia sensor [8]. A fabric-compatible UV sensor was presented using a cellulose-based thread coated with single-walled carbon nanotube ink [9]. An immunosensor for dengue virus (viral protein 1, NS1) detection with an excellent detection limit (in the order of 12 ng mL^−1^) and a sensitivity of 85.59 μA mM^−1^ cm^−2^ based on a CNT screen-printed electrode was developed [10].

There are some key issues in the application of nanomaterial-based conductive inks. SWCNTs face challenges for use in inks due to their low dispersity in most solvents and the contradictory reports on their cytotoxicity. The applied surfactants are sodium dodecyl sulfate salt (SDS) or silk sericin [3]. A mixture of sodium dodecylsulphonate (SDBS) and hexadecyl trimethylammonium bromide (CTAB) as surfactants was used to prepare homogenous SWCNT ink and, further, to form transparent conducting SWCNT films by using “Mayer rod coating” at room temperature [11].

The other crucial issue is the good adhesion of nanotube ink to selected surfaces.

Despite these difficulties, CNTs characterized by excellent electrical and mechanical properties are ideal candidates for flexible wearable devices [11]. On the other hand, the most suitable base for designing these kinds of tools is textile structures. Fiber-based materials are expected to be flexible and stretchable. Compared with rigid and planar electronic devices, fiber-based wearable electronics provide significant advantages in terms of flexibility, stretchability and breathability, and they are considered the pioneers in the new generation of soft wearables [12].

Amongst the variety of textiles proposed for wearable electronics, silk-based materials are the most promising due to their low cytotoxicity, their biocompatibility and biodegradability, and the fact that they do not cause inflammation and skin irritation [13].

In this study, silk fabric, a natural product made of silk fibroin, was selected as a non-toxic material. Silk fabric was functionalized with commercial SWCNT ink with SDS as an efficient surfactant to disperse SWCNTs. SWCNT ink was applied by using the dip-coating method, which is a new approach. Moreover, SWCNT ink was not applied directly on the silk fabric, which is the usual practice. Silk fabric was initially coated with polydopamine (PDA) in the process of direct polymerization on the textile surface. PDA is characterized by many functional groups, enabling the efficient immobilization of SWCNTs and the improvement in their chemical bonding to the silk fabric. This new approach allows us to significantly lower the electrical percolation threshold in comparison to other reports.

## 2. Results and Discussion

To make silk fabric electroconductive, SWCNT ink was applied. The silk fabric was previously coated with polydopamine (PDA, sample labeled as silk_PDA) so SWCNTs would bind efficiently to the silk surface. The procedure of polymerization of dopamine to PDA, rich in functional groups to bind with various molecules and materials, was described in detail in our previous report on multifunctional silk fabric modified with silver nanowires by using PDA as a linker [14]. PDA contains functional groups, such as catechol groups, aromatic groups, amines and imines, and provides a large number of active sites through strong chemical interactions such as hydrogen bonding, electrostatic interactions, π-π stacking, coordination or chelation [15].

A PDA/CNT nanocomposite was previously obtained by functionalization with dopamine through a reaction between the hydroxyl groups of CNTs and dopamine [16]. The PDA/SWCNT composite and subsequent modification with PEG creates a versatile platform with radionuclide labeling for multimodal tumor imaging and therapy [17].

**Figure 1:**
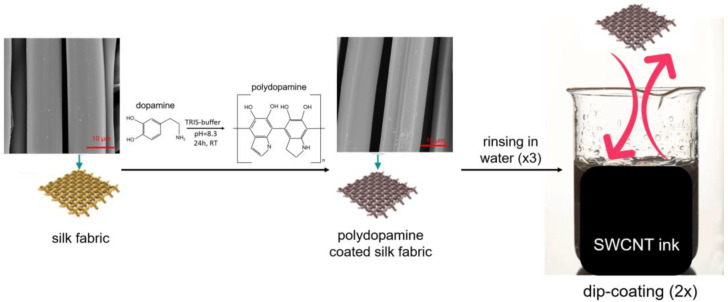
A schematic image of the subsequent stages of the silk fabric functionalization process.

In this study, silk fabric, after being coated with PDA, was immersed in diluted aqueous SWCNT ink (samples labeled as SWCNT0.01 to SWCNT0.21, Table 1). According to the safety data sheet of the ink, it contains a surfactant of SDS, a commonly used dispersing agent for obtaining homogenous carbon-based nanomaterial solutions and preventing their agglomeration [18]. Figure 1 shows a schematic image of the subsequent stages of the silk fabric functionalization process. The samples were weighed before and after functionalization with SWCNT ink to obtain a value of SWCNT ink deposition on the silk fabric in g/m^2^ (Table 1). A total of 0.2 mg of SWCNT was in 1 mL of the ink. The ink was also weighed after water evaporation which made it possible to calculate the nominal SWCNT weight percentage in the ink and also on the silk fabric. 

**Figure 2:**
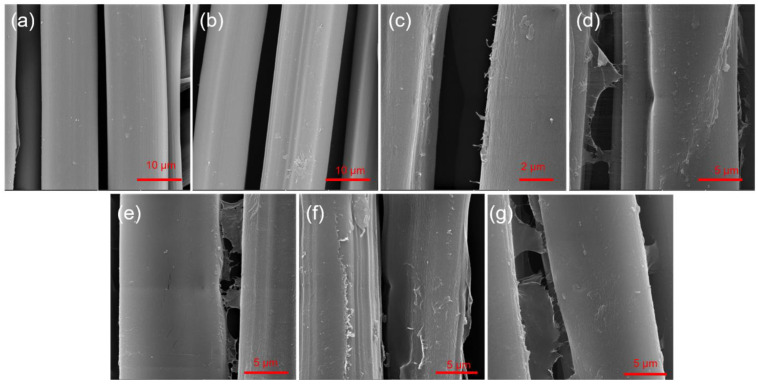
SEM images of (**a**) pure silk fabric, (**b**) silk fabric functionalized with PDA, and silk fabric coated with PDA with different concentrations of SWCNTs: (**c**) SWCNT0.01, (**d**) SWCNT0.02, (**e**) SWCNT0.12, (**f**) SWCNT0.17 and (**g**) SWCNT0.21.

Figure 2 shows SEM images of the silk fibers. The morphology of pure silk fibers (Figure 2a) and then after PDA coating (Figure 2b) does not change significantly, indicating the presence of a thin layer of PDA. The fibers after SWCNT coating reveal protruding nanotubes on the edges and plates of SWCNT ink on the silk fiber surface and between the fibers (Figure 2c–g).

The chemical composition of the fabric changes after SWCNT ink functionalization due to the surfactant’s presence (Table 2). The main element of the fabric as well as of the SWCNTs is carbon, but the SDS included in the ink has, besides carbon and oxygen, sodium and sulfur. The weight percentage of sodium and sulfur increases from the SWCNT0.01 to SWCNT0.21 samples due to an increase in SWCNT ink deposition on the silk fabric. The pure silk fabric contains a trace amount of sulfur and sodium. SF consists of a heavy (H) chain (~390 kDa) and a light (L) chain (~26 kDa) linked together by a single disulfide bond, and the presence of sulfur indicates natural silk [19]. The trace amount of sodium (Na) is typical for silk fabric treated with sodium carbonate as a degumming agent [20].

**Figure 3:**
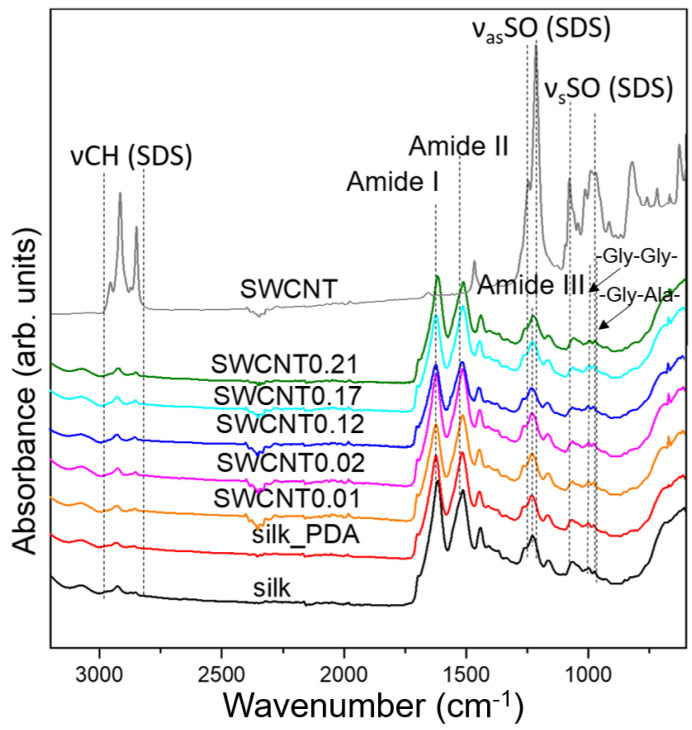
IR spectra of pure silk fabric, silk fabric functionalized with PDA and silk fabric functionalized with PDA and with different concentrations of SWCNTs. SWCNT ink is labeled as SWCNT.

Infrared spectroscopy was applied to study the structure and conformation of silk fibroin (Figure 3). The main bands typical for silk fibroin (SF) in terms of protein amide I, amide II and amide III appear at 1618, 1512 and 1265 cm^−^^1^, respectively [20]. The presence and intensity of these bands indicate the dominant β-sheet structure of the protein. The amorphous part is also present as a band at 1225 cm^−^^1^, responsible for the random coil structure of amide III [21]. Moreover, a typical raw silk broad band at about 3270–3280 cm^−1^ indicates a lack of sericin, with a more random structure compared to high-crystalline fibroin with stacked β-sheets [22]. The sericin is removed from the cocoons in the degumming process, as one of the main stages of silk textile fabrication [19]. Moreover, the structures without the sericin show two low-intensity bands at 974 and 995 cm^−^^1^ related to the abundance of glycine–alanine and glycine–glycine fragments in the polypeptide chain, characteristic of SF [22]. The IR results confirm that the degumming process was carried out efficiently due to the lack of sericin in the structure.

The IR spectrum of the SWCNT ink shows several intense bands in the region from 2850 to 3000 cm^−^^1^ due to the CH stretching of CH_2_ and CH_3_ groups in SDS, as follows: at 2956 cm^−^^1^ due to CH_3_ asymmetric stretching, at 2916 cm^−^^1^ due to CH_2_ asymmetric stretching and at 2850 cm^−^^1^ due to CH_2_ symmetric stretching. There is also a characteristic, sharp, intense band at 1217 cm^−^^1^, which is related together with the other lower intensity band at 1246 cm^−^^1^ to the asymmetric stretching vibration of the SO bond in SDS [23]. Two bands at 981 and 1080 cm^−^^1^ are assigned to the symmetric stretching of the SO bond in SDS [24].

The IR spectra of the silk fabric after functionalization with PDA and different amounts of SWCNT ink reveal similar IR spectra to those of pure silk (Figure 3) and indicate that the functionalization process does not damage the structure of silk fibroin.

**Figure 4:**
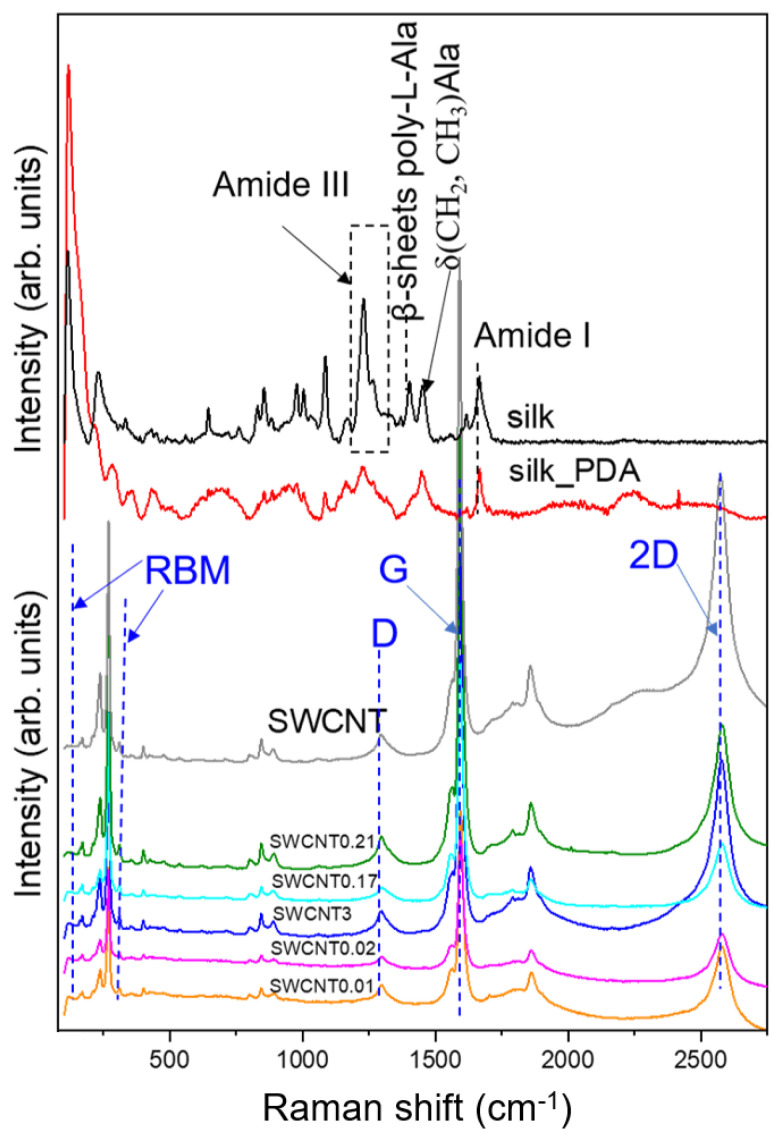
Raman spectra of pure silk fabric, silk fabric functionalized with PDA and silk fabric functionalized with PDA and with different concentrations of SWCNTs. SWCNT ink is labeled as SWCNT.

Raman spectroscopy was implemented in this study as a complementary technique to IR spectroscopy, mainly to evaluate the SWCNT assignments not visible in the infrared spectra. Raman spectroscopy analysis revealed typical characteristics of silk fibroin in the silk fabric (Figure 4). The main peak typical for SF at 1666 cm^−^^1^ is related to the C=O stretching of amide I and confirms the dominant β-sheet structure of the fibroin. The β-sheet structure of SF is apparent in the spectra also due to the presence of amide III bands at 1230 and 1265 cm^−^^1^, respectively, related to stacked β (high intensity) and disordered β-sheets (low intensity indicating low contribution) [20]. The other band originated from the β-sheet structure and is associated with poly-L-alanine (Ala) regions at 1401 cm^−^^1^ and has a higher intensity than a band at 1453 cm^−^^1^ related to CH_2_ and CH_3_ bending in Ala. The above characteristic bands for SF cannot be distinguished in the silk fabric covered with SWCNT ink due to the high intensity of the Raman signal from the nanotubes and the PDA influence. The PDA layer on textile structures significantly decreases the textile’s band intensities or even makes them not apparent in Raman spectra [14]. This also confirms the efficient functionalization of textiles by using PDA.

The SWCNT ink spectrum shows typical bands for carbon nanotubes (Figure 4) [25]. The G band at around 1580 cm^−^^1^ is related to sp^2^-hybridized carbon atoms in the nanotube wall. The characteristic for the SWCNT “G” band has two peaks, the stronger one at 1590 cm^−^^1^ associated with vibrations of carbon atoms along the nanotube axis, and the smaller one at 1565 cm^−^^1^ with the vibrations along the circumference of the SWCNT. The other typical “D” band centered at 1300 cm^−^^1^, known as the disorder band or the defect band, is related to nanotube quality. The D band is activated in the first-order scattering process of sp^2^ carbons by the presence of in-plane substitutional hetero-atoms, vacancies, grain boundaries or other defects and by finite size effects [26]. 

The intensity ratio of D and G (I_D_/I_G_) bands is considered a unique characteristic tool to measure the density of defects and ascertain the degree of graphitization [27]. The I_D_/I_G_ ratio of the SWCNTs in the ink was calculated to be about 0.07, indicating a high-quality carbon nanomaterial with a slight amount of defects. After coating the silk fabric functionalized with PDA with the ink, the I_D_/I_G_ ratio of the SWCNTs increased to about 0.34, indicating the completion of the functionalization process. The increase in the D-band intensity and I_D_/I_G_ ratio of SWCNTs after functionalization can be attributed to carbon atoms excited from sp^2^ to sp^3^ hybridization [28].

The resonant “2D” band at 2600 cm^−^^1^ is an overtone of the “D” band. Another sign of SWCNT presence is the group of bands between 200 and 300 cm^−^^1^, called the radial breathing modes (RBMs) of SWCNTs. RBMs allow us to determine the SWCNTs’ diameter as well as their chirality.

**Figure 5:**
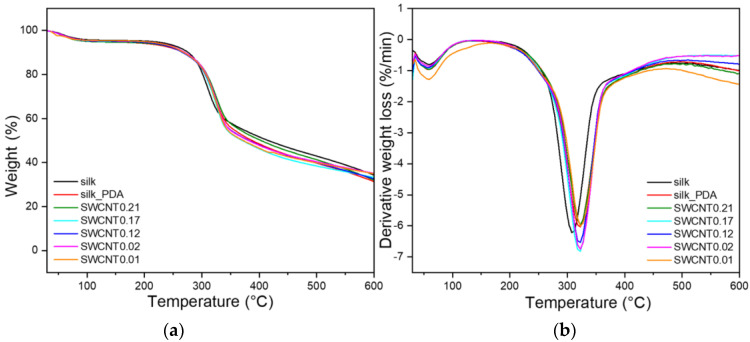
TG (**a**) and DTG (**b**) thermograms of pure silk fabric, silk fabric functionalized with PDA and silk fabric functionalized with PDA and with different concentrations of SWCNTs. SWCNT ink is labeled as SWCNT.

Figure 5 shows the thermogram of the silk fabric with the main peak at 304 ºC, which is related to the thermal decomposition of silk fibroin [20]; the peak was shifted to 323 ºC after PDA functionalization (Figure 5). The same phenomenon was observed for the silk fabric coated with PDA, which was used as a linker for the bonding of silver nanowires [14]. The thermal decomposition peak for the PDA and SWCNT ink-coated fabric also shows the maxima at 323 °C, similar to the fabric functionalized only with PDA. The thermal decomposition process also started at higher temperatures, at 263 °C for the silk fabric, while for the silk_PDA sample, it was at 286 °C. The silk fabric after PDA functionalization revealed slightly higher resistance to heat compared to the pure silk fabric, and after SWCNT ink functionalization, this higher resistance to heat was retained. SWCNTs are heat-resistant and do not decompose in this temperature range. SWCNTs start to melt at 5100 K (4827 °C) [29]. It is found that SWCNTs are thermally more stable than multi-walled CNTs [30]. SWCNTs are one of the most thermally and mechanically resistant nanomaterials. Due to their remarkable physical, chemical, mechanical (Young modulus from 1 to 5 TPA; tensile strength from 11 to 63 GPa) and thermal properties, they are selected as a filler for many applications, even for use in harsh and difficult conditions [29].

**Figure 6:**
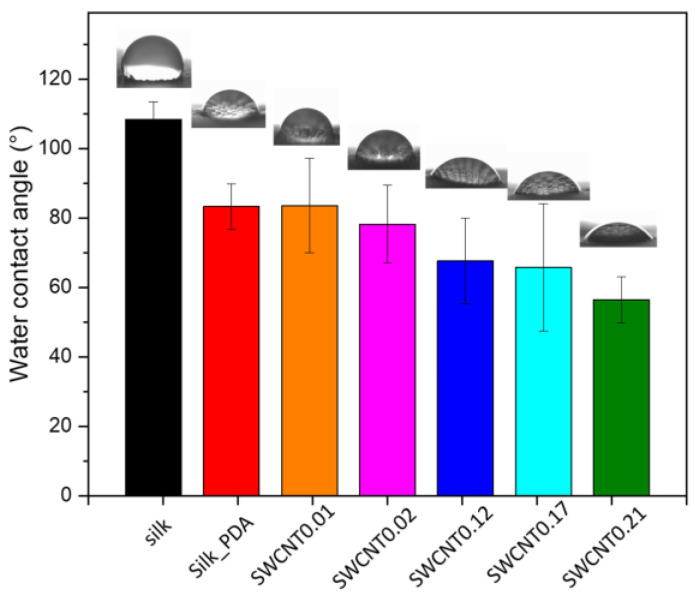
Water contact angle of pure silk fabric, silk fabric functionalized with PDA and silk fabric with PDA and with different concentrations of SWCNTs.

The functionalization of the silk fabric modified its wettability (Figure 6). The water contact angle of the pure silk fabric was determined to be about 108°, whereas after PDA functionalization, the silk fabric became hydrophilic (83°). PDA is known to decrease the water contact angle of hydrophobic surfaces and make even a superhydrophobic surface hydrophilic [31]. The water contact angle decreased with the increasing amount of SWCNT ink deposited on the silk fabric surface, as follows: 83.5°, 78°, 67°, 65° and 56° for SWCNT0.01, SWCNT0.02, SWCNT0.12, SWCNT0.17 and SWCNT0.21, respectively. The water contact angle decreased due to the surfactant of SDS presence and the increase in its concentration as a result of the increase in the ink deposition. The EDS data (Table 1) are consistent with the wettability results and show the increase in SDS content on the silk fabric with the increase in the ink concentration. The EDS results are not able to confirm the first step of the functionalization process and the formation of the PDA layer due to the chemical similarity of the silk fabric and PDA. However, without the PDA influence and the formation of a thin layer on the silk fibers, the transition of the fabric from hydrophobic to hydrophilic would not be possible. SDS is an amphiphilic molecule with both types of groups, hydrophilic and hydrophobic, and is highly soluble in water. SDS disperses CNTs in aqueous solutions mainly through hydrophobic/hydrophilic interactions, in which the hydrophobic tail of a surfactant molecule adsorbs on the surface of CNT bundles, while its hydrophilic head associates with water for dissolution. It was shown that the water contact angle between hydrophobic/superhydrophobic surfaces and SDS solutions continuously decreases as the SDS concentration increases and then becomes constant when the SDS concentration reaches the critical micelle concentration (CMC) [32]. 

Inks with conductive nanomaterials are used to make surfaces electroconductive. The crucial issue in making materials, including textiles, electroconductive is the amount of applied ink and the content of nanomaterial in the ink. According to the percolation theory, the level of the conductive nanomaterial, called the filler, should be low to facilitate the method and lower the cost of making the material electrically conductive. This level determines the percolation threshold, the level at which the textiles convert from an insulator to a conductor due to the formation of a continuous conducting network. 

Below the percolation threshold, the electrical properties are determined by the dielectric matrix because the fillers do not form a continuous network for electrons to flow within the fabric. When the filler content increases, SWCNT ink facilitates the formation of conductive paths through the interaction between SWCNTs and significantly changes the electrical properties of the fabric.

**Figure 7:**
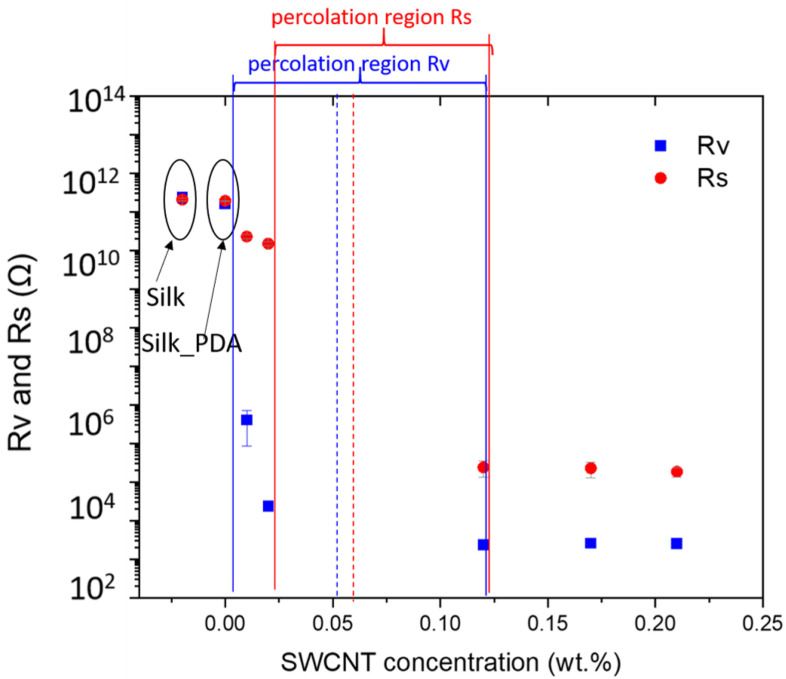
Electrical surface and volume resistance of pure silk fabric, silk fabric functionalized with PDA and silk fabric with PDA and with different concentrations of SWCNTs.

To study the electroconductivity of the silk fabric after functionalization dependent on SWCNT content, electrical volume (Rv) and surface resistances (Rs) were measured according to the standards [33,34] (Figure 7). In these standards, resistances are used for the characterization of electrical properties of textile structures. For this reason, the percolation region in Figure 7 is presented in value (resistance) and unit (Ω), typical for the evaluation of electrical properties of textiles, which allows for a comparison of the results for different textile structures and applied functionalization systems.

Volume and surface resistances for the pure silk fabric were measured to be about 2.4 × 10^12^ Ω and 2.1 × 10^12^ Ω, respectively. PDA functionalization did not significantly modify the electrical properties of the silk fabric, with volume and surface resistances of 1.6 × 10^12^ Ω and 1.9 × 10^12^ Ω, respectively. PDA did not induce conductive properties and did not have the ability to significantly change the electrical surface and volume resistances [35]. 

The application of SWCNT ink on PDA-coated silk fabric changed the electrical properties of the fabric. Volume resistance values determine an insulating zone for SWCNT concentration. The volume resistance value for the lowest applied concentration of SWCNT of 0.01 wt.% was (4.1 ± 0.3 × 10^6^ Ω), for 0.02 wt.% of SWCNT, the resistance decreased by two more orders of magnitude (2.3 ± 0.4 × 10^4^ Ω), and for 0.12 wt.%, it additionally decreased by one order of magnitude (2.3 ± 0.4 × 10^3^ Ω). For higher concentrations of SWCNT, the volume resistance did not change significantly and was at the level of 10^3^ Ω, determining the conductive zone of the composites.

The surface resistance values indicate an insulating zone for SWCNT concentrations below 0.02 wt.% (1.5 ± 0.2 × 10^11^ Ω); for 0.12 wt.% of SWCNT, the resistance decreased by six orders of magnitude (2.4 ± 0.1 × 10^5^ Ω), and for 0.17%wt. and above concentrations of 0.21 wt.%, it remained at the level of 10^5^ Ω; 2.3 ± 0.9 × 10^5^ Ω and 1.8 ± 0.5 × 10^5^ Ω, respectively.

The surface and volume resistances are influenced by SWCNT ink, but not in the same way.

The percolation threshold was slightly lower for the volume resistance, at about 0.05 wt.%, than for the surface resistance, at about 0.06 wt.% A similar phenomenon was observed for silk fabric functionalized with silver nanowires (AgNWs). The volume resistance values were smaller in comparison to the surface resistance values for the same amount of AgNWs applied for functionalization due to the fabric structure, which allows for the incorporation of nanostructures within the fabric structure between single silk fibers and facilitates the formation of percolation paths for electron flow [14]. These results are related to the structure of the fabric. Surface resistance allows for measurements of the fibers on the top part of the textile structure and is influenced by the percolation paths formed on the fabric surface. The fabric is composed of entwined fibers, and in the case of volume resistance measurements, all of the silk fiber surfaces covered with SWCNT ink throughout the fabric (0.2 mm thick) have an impact on the resistance value. The internal structure within the fabric is extensive and many percolation paths are formed inside the fabric. This results in lower values of volume resistance and a lower value of the percolation threshold in comparison to surface resistance.

Both percolation threshold values are low, indicating that a very low content of SWCNTs makes silk fabric electroconductive. These results also demonstrate the high quality of the ink. In comparison to other reports, which show a low percolation threshold, this is a significantly lower value. The electrical percolation threshold was about 4 wt.% of SWCNT loading in polyethylene composites prepared by using the melt processing technique [36]. The percolation threshold of carbon nanotube-based epoxy nanocomposites with a thickness of 0.5 mm was reported as 0.17 wt.% [37]. For polystyrene loaded with SWCNTs, using a mixture of conductive polymers, poly(3,4-ethylenedioxythiophene)/poly(styrene sulfonate) (PEDOT/PSS), as surfactants instead of SDS allowed the researchers to obtain a lower percolation threshold value of 0.2 wt.% [38]. The percolation threshold values were also lower in comparison to other additives, such as AgNWs, which are covered with PVP, a capping agent applied during polyol synthesis. PVP impairs the formation of percolation paths [14]. AgNWS are also characterized by low atmospheric stability and corrosion due to sulfidation and oxidation processes [39], while SWCNTs are characterized by high environmental stability [3]. Most electronic applications use MWCNTs rather than SWCNTs due to their high adhesion to substrates [38]. MWCNTs have more active sites at the end of the tube, but in this study, this key issue was overcome by means of PDA application. Moreover, SWCNTs are more expensive to produce than MWCNTs [40]. The lower the percolation threshold, the lower the amount of SWCNTs needed. By lowering the value of the percolation threshold, the price of SWCNT-based ink is also lowered. 

In this report, the volume and surface resistance thresholds of about 0.05 wt.% and 0.06 wt.%, respectively, indicate the efficient process of functionalization, which needs only a small amount of SWCNT ink to obtain highly electroconductive textiles. The ink was optimized productively and the PDA influence also played a crucial role in the process of efficient functionalization.

## 3. Materials and Methods

### 3.1. Functionalization of Silk Fabric with SWCNT Ink

Pure silk woven fabric characterized by a mass per unit area of 70 g/m^2^, a linear mass of weft and warp yarns of 37 dtex, and a thickness of 0.2 mm was obtained from Łukasiewicz-Textile Research Institute resources. Single-walled carbon nanotube (SWCNT) conductive aqueous ink containing 0.2 mg of SWCNTs per 1 ml of the ink was purchased from Merck (791490, <400 Ω/sq). 

The silk fabric was cut into 10 cm × 10 cm pieces and immersed in a dopamine hydrochloride (2-(3,4-dihydroxyphenyl)ethylamine hydrochloride, Fluorochem) solution for 24 h. The solution was prepared by dissolving 2 g of dopamine hydrochloride in 1 L of distilled water and stirring the mixture for 1 h; then, Tris/glycine buffer (25 mM Tris and 192 mM glycine, pH 8.3, Bio-Rad) was added to the dopamine hydrochloride solution at a ratio of 1:10 (*v*/*v*). After 24 h, the samples were rinsed three times in deionized water and dried. The polydopamine (PDA)-coated samples were dipped in SWCNT ink for 10 s and dried again. The ink was diluted in distilled water to obtain different concentrations of SWCNTs and to allow for coating with different amounts on the silk fabric. To SWCNT ink, different amounts of water were added at volumes of 1:7, 1:3, 1:1.6, 1:1 and 1:0.6, and the obtained solutions were characterized by increasing concentration of SWCNTs. The silk fabric was dip-coated in the above-diluted solutions twice for each sample. The samples immersed in a solution of the lowest concentration of SWCNTs were labeled as SWCNT0.01, and the name of the samples changed from SWCNT0.01 to SWCNT0.21 (Table 1) according to the increasing concentration of SWCNTs in the solutions in which the samples were immersed. After the functionalization process, the samples were dried at room temperature to evaporate the water. 

### 3.2. Characterization of Pure Silk Fabric and Silk Fabric Functionalized with SWCNTs

The sample surface was visualized using SEM (VEGA 3 TESCAN, Company, City, Czech Republic) at an accelerating voltage of 20 kV. The samples for SEM analysis were covered with a thin gold layer of about 3 nm. Chemical composition was investigated through EDS analysis (INCA energy X-ray energy dispersion spectrometer, Oxford Instruments, Abingdon, UK) of three different areas of 0.2 mm^2^, with an accelerating voltage of 20 kV and a pressure of 20 Pa (X-ray microanalyser INCA Energy, Oxford Instrument Analytical). The statistical analysis for the EDS measurements was performed using the INCA energy software (version 4.15). Raman spectra of the samples were obtained by using an inVia Renishaw Raman Microscopy System (Renishaw, Wotton-under-Edge, UK) with a 50× microscope objective (LEICA, Wetzlar, Germany). The excitation source was a laser with a wavelength of 785 nm. Each Raman spectrum was obtained with four accumulations in the range from 600 to 2000 cm^−1^ corrected by the WiRE™5.3 software. The Raman spectra were presented without normalization. The analysis of the band intensity was based on the comparison of selected bands within one spectrum and then this intensity ratio was compared for all samples. The Fourier transformed infrared (FTIR) absorption spectra of the silk samples were recorded using a BRUKER Vertex 70 FTIR spectrometer with a diamond ATR (Bruker, Bremen, Germany) spectrometer in a spectral range from 600 cm^−1^ to 4000 cm^−1^ with a resolution of 4 cm^−1^. The Raman and infrared spectra were collected in at least three replicates for each sample.

The thermal properties were studied using the TG/DTG (TG 209F1 Libra, Netzsch, Selb, Germany) technique. The thermal measurements were performed at least three times for each sample. The samples with a weight of about 4 mg were placed in a ceramic crucible with a volume of 85 μL and heated at a rate of 10 °C min^−1^ under a nitrogen flow of 25 mL min^−1^ in the temperature range of 20–800 °C. The water contact angle (θ) was analyzed using a goniometer PGX (Fibro System AB). Water was automatically applied at a constant volume of 4 μL (±0.2 µL) to the tested surface with the goniometer. At least ten measurements were taken for each sample.

The electrical resistance of the silk fabrics before and after functionalization with graphene was measured according to the standards [33,34] using a 6206 thermometer (ELTEX) and standardized electrodes. The electrical surface (Rs) and volume (Rv) resistances of the samples were measured after 24 h of conditioning in an air-conditioned HCZ 0030 L(M) chamber (Heraeus) at a temperature of 23 (±2) °C and a relative humidity of 25 (±5)%. The samples were measured in the same, above-described conditions.

## 4. Conclusions

This study proposes electroconductive silk fabric functionalized with SWCNTs for future potential designs of smart textiles. Silk fabric is formed of silk fibroin protein with high biocompatibility, low cytotoxicity and biodegradability. Silk fabric was first coated with PDA through direct polymerization on the fabric. PDA, characterized by many functional groups, facilitates the immobilization of carbon-based nanomaterials on the silk surface. Efficient immobilization of SWCNT ink due to PDA moieties allows for the application of less amount of ink on the silk fabric to obtain high electrical conductivity. The silk/PDA fabric was functionalized with SWCNT ink of different concentrations of SWCNTs by using the dip-coating method. PDA improves the adhesiveness of SWCNTs to silk fabric. 

The volume and surface resistance thresholds were determined to be about 0.05 wt.% and 0.06 wt.%, respectively. The low value of the percolation threshold indicates the efficient process of functionalization, with the high-quality ink facilitating the formation of percolation paths through SWCNTs. SWCNT ink containing SDS is well optimized to obtain a homogenous ink.

The functionalization process does not change the dominant β-sheet structure of silk fibroin according to IR and Raman spectroscopies. Functionalization slightly improves the heat resistance and significantly decreases the water contact angle. The silk fabric is converted from hydrophobic to hydrophilic due to the influence of PDA and SDS, which act as a surfactant of SWCNTs.

SWCNT ink-coated silk fabric of high electrical conductivity offers a novel building block for further designing devices and products for wearable electronic and biomedical applications.

## Figures and Tables

**Table 1:** Sample names and description of silk fabric functionalized with PDA and SWCNT ink.

Sample Name (Sample Coated with PDA and SWCNT Ink)	Solution for Functionalization, SWCNT Ink/Water (*v*/*v*)	SWCNT Ink Deposition on Silk Fabric (g/m^2^)	Nominal SWCNT Deposition on Silk Fabric (mg/m^2^)	Nominal SWCNT on Silk Fabric (wt.%)
SWCNT0.01	1:7	0.44 ± 0.02	9	0.01
SWCNT0.02	1:3	0.71 ± 0.03	14	0.02
SWCNT0.12	1:1.6	3.65 ± 0.08	73	0.12
SWCNT0.17	1:1	5.57 ± 0.05	111	0.17
SWCNT0.21	1:0.6	6.87 ± 0.12	137	0.21

**Table 2:** Results of EDS analysis of pure silk fabric, silk fabric functionalized with PDA, and silk fabric with PDA and with different concentrations of SWCNTs.

	C (wt.%)	N (wt.%)	O (wt.%)	Na (wt.%)	S (wt.%)
Silk	48.12 ± 0.14	20.97 ± 0.22	30.51 ± 0.14	0.32 ± 0.01	0.08 ± 0.01
Silk_PDA	48.39 ± 0.03	20.94 ± 0.16	30.60 ± 0.13	0.00	0.07 ± 0.01
SWCNT0.01	48.68 ± 0.19	20.59 ± 0.32	30.47 ± 0.26	0.09 ± 0.01	0.18 ± 0.00
SWCNT0.02	48.78 ± 0.12	20.67 ± 0.06	30.21 ± 0.10	0.12 ± 0.00	0.22 ± 0.01
SWCNT0.12	48.99 ± 0.22	20.10 ± 0.15	30.34 ± 0.20	0.23 ± 0.02	0.34 ± 0.02
SWCNT0.17	48.80 ± 0.28	20.17 ± 0.27	30.33 ± 0.07	0.28 ± 0.01	0.42 ± 0.03
SWCNT0.21	48.97 ± 0.23	19.77 ± 0.35	30.47 ± 0.13	0.33 ± 0.01	0.46 ± 0.02

## Data Availability

The data presented in this study are available on request from the corresponding author.
